# Evaluation of cinnamaldehyde mucoadhesive patches on minor recurrent aphthous stomatitis: a randomized, double-blind, placebo-controlled clinical trial

**DOI:** 10.1186/s12903-022-02248-5

**Published:** 2022-06-14

**Authors:** Tahere Molania, Ali Malekzadeh Shafaroudi, Majid Saeedi, Mahmood Moosazadeh, Faeze Valipour, Seyyed Sohrab Rostamkalaei, Negareh Salehabadi, Maede Salehi

**Affiliations:** 1 Department of Oral Medicine, Faculty of Dentistry, Mazandaran University of Medical Sciences, Sari, Iran; 2 Dental Research Center, Mazandaran University of Medical Sciences, Sari, Iran; 3 Student Research Committee, Faculty of Dentistry, Mazandaran University of Medical Sciences, Sari, Iran; 4 Department of Pharmaceutics, Faculty of Pharmacy, Mazandaran University of Medical Sciences, Sari, Iran; 5 Gastroinstitional Cancer Research Center, Non-Communicable Diseases Institute, Mazandaran University of Medical Sciences, Sari, Iran; 6 Mazandaran University of Medical Sciences, Sari, Iran; 7 Department of Pharmaceutics, Faculty of Pharmacy, Ayatollah Amoli Branch, Islamic Azad University, Amol, Iran; 8 Medicinal Plant Research Center, Faculty of Pharmacy, Ayatollah Amoli Branch, Islamic Azad University, Amol, Iran

**Keywords:** Recurrent aphthous ulcer, Cinnamaldehyde, Treatment

## Abstract

**Background & objective:**

The use of herbal medicines to treat common oral diseases increases rapidly. Recurrent aphthous stomatitis is one of the most common oral mucosal diseases, which has an unclear etiology and could lead to severe pain and dysfunction. Cinnamaldehyde is a major component of cinnamon bark oil. Biological properties of cinnamaldehyde, such as antioxidant, antitumor, antifungal, cytotoxic, and anti-mutational characteristics, have been identified. Considering the prevalence of recurrent aphthous stomatitis and the importance of using herbal resources for treatment, the present study aimed to evaluate the effect of mucosal adhesive patches containing Cinnamaldehyde on minor recurrent aphthous stomatitis lesions.

**Material & methods:**

In this randomized, double-blind clinical trial, patients were divided into two groups. The intervention group received three daily mucosal adhesive patches to be used in the morning, afternoon, and night. The control group also did the same with a placebo. To evaluate the healing and determine the diameter of the lesions, patients were clinically examined on days zero, 3, 5, and 7. The VAS scale evaluated pain at baseline and after each meal for seven days. The Fisher's exact test, t-test, Shapiro Wilk test, Friedman test, and the Mann–Whitney test were used to analyze the data using the SPSS 20 software.

**Results:**

There was no statistically significant difference in the mean diameter of the inflammatory lesion and pain intensity in the two groups in the baseline (*p* > 0.05). However, the ulcer size was significantly reduced in the cinnamaldehyde group on the third, fifth, and seventh days of the study. Except for baseline, the mean pain intensity significantly decreased in the cinnamaldehyde group compared to the placebo group (*p* < 0.05).

**Conclusion:**

Cinnamaldehyde mucoadhesive patches effectively reduced and improved aphthous lesions and pain intensity in patients and can be considered a treatment for RAS. Registration number: IRCT20180312039060N2—First registration date: 20/07/2018. The present study was registered as a retrospective study.

## Introduction

Recurrent aphthous stomatitis (RAS) is one of the most common diseases of the oral mucosa. The prevalence of this lesion varies between 5 and 25% and occurs more often in individuals between the ages of 10 and 40 [1, 2]. Aphthous lesions are circular or elliptical-shaped lesions with a specific margin covered with a white–gray membrane surrounded by an erythematous halo that could be extremely painful [2]. Aphthous lesions involve the non-keratinized mucosa, particularly mucosa of lips, cheeks, soft palate, lateral and ventral surfaces of the tongue, and the floor of the mouth [3].

RAS is classified into three minor, major and herpetic forms based on clinical appearance (size and shape of the ulcers). The etiology of aphthous lesions is unknown; however, some factors are believed to be involved in its occurrence, including genetic factors, food allergens, superficial trauma, endocrine changes (menstrual cycle), stress, anxiety, and smoking cessation, some chemicals, and microbial agents [4].

These factors can directly or indirectly alter the oxidant / antioxidant balance of the body and accelerate the production of free radicals. A free radical is commonly an atomic or molecular sample with one or more unpaired electrons in its structure [5]. The most important free radicals in the biological system are oxygen-based radicals. Other reactive compounds are known as Reactive Oxygen Species (ROS) [6]. Oxidative stress occurs when the intracellular concentrations of ROS are higher than the physiological levels produced inside or outside the cell [7]. Increased oxidative stress and oxidant/antioxidant imbalance are essential for inflammatory reactions of oral aphthous lesions. Conditions such as viral and bacterial infections, hyperthermia, and UV radiation can result in oxidative stress [8].

Different treatments are used to heal oral aphthous ulcers, including 1- local anesthetics such as Lidocaine spray or gel that alleviate symptoms and reduce the healing period. 2- anti-inflammatory and antiseptic medications such as mouthwashes containing chamomile extract or chlorhexidine. 3- Topical corticosteroids such as Triamcinolone, and 4- Topical treatments with Tetracycline that alleviate the pain and reduce the healing period [9]. Other therapeutic methods have also been evaluated, including toothpaste containing specific enzymatic compounds that reduce aphthous attacks and alleviate pain [10].

For centuries, treatment with medicinal herbs has been the only source available to numerous groups and tribes. Today, plants are used in complementary medicine to treat, reduce or prevent many diseases [11]. Recently, the attention given to herbal medications and their traditional use has increased globally [12]. Many patients tend to use herbal medicine regarding the side effects of chemical drugs.

Some studies have reported the effectiveness of Chamomile and Aloe Vera in the treatment of recurrent aphthous stomatitis as flavonoid compounds in these plants reduce the severity of ulcers with their anti-inflammatory effects [13]. In another study, Myrtus communis effectively reduced the pain, ulcer size, and erythema in patients with aphthous ulcers and improved their quality of life [14]. Cinnamomum Cassia is an evergreen tree in southern China and is widely cultivated in other parts of Asia in the south and east Asia (India, Indonesia, Laos, Malaysia, Taiwan, Thailand, and Vietnam) [15]. It has traditionally been used to treat erectile dysfunction, gastritis, blood circulatory disorders, and inflammatory diseases [16]. Cinnamaldehyde is an aromatic aldehyde compound and the main ingredient of cinnamon oil (about 65%), widely used as a flavoring agent in foods and beverages such as ice cream, sweets, and chewing gum [17, 18]. The biological properties of cinnamon oil, such as peripheral vasodilatory, antitumor, antifungal, cytotoxic, and anti-mutation characteristics, are mainly attributed to cinnamaldehyde [19–21]. However, despite the various biological activities of cinnamaldehyde, its mechanism for preventing cell growth is currently unknown. Some studies suggest that Cinnamaldehyde prevents the growth of microorganisms using its aldehyde structure [22]. Cinnamaldehyde inhibits IL-1β and TNF-α in macrophages and reduces the active oxygen species (ROS). It also reduces the phosphorylation of protein kinase stimulated by lipopolysaccharides [23]. Considering the prevalence of recurrent aphthous stomatitis and the importance of utilizing herbal resources to treat diseases, this study aimed to treat patients with minor recurrent aphthous stomatitis by developing a Cinnamaldehyde mucoadhesive patch.

## Materials and methods

### Research design and ethical considerations

The Medical Ethics Committee of Mazandaran University of medical sciences approved this randomized, double-blind clinical trial (Moral code: IR.MAZUMS.REC.1396.10438). All patients entered the study after receiving adequate explanations about the treatment of the disease, possible complications, the therapeutic process, and signing the consent form. The present study was registered as a retrospective study.

### Participants and selection criteria

Based on the study of Babaei et al., with a mean and standard deviation of the ulcer diameter on the 7th day, 1.29 ± 0.66 in the interventional group and 0.60 ± 0.69 in the control group, a confidence level of 95%, a test power of 90%, and for the two-way test, by using the formula for comparing the two means in the G-power software. The sample size of the present study was determined to be 44 patients (22 patients in each of the interventional and control groups) [13].

Patients with recurrent aphthous stomatitis aged 20–40 years who were referred to the Oral Diseases Department of Sari dental clinic in October 2018 and had a history of minor aphthous ulcers in their lips and buccal mucosa were selected based on the inclusion criteria of the study. Patients were randomly allocated to the intervention group (*N* = 22) or the control group (*N* = 22). The head nurse of the medical center (someone other than the analyzers and evaluators) registered the participants and gave them medicine (cinnamaldehyde mucoadhesive tablets or placebo). The duration of the intervention was seven days.

The inclusion criteria of this study were patients who had minor recurrent aphthous stomatitis in their lips and buccal mucosa, no systemic diseases, no use of immunosuppressive medicines for at least one month, and no use of dentures and antibiotics. Also, pregnant women, who could not use mucoadhesives patches, patients with syndromes that aphthous-like lesions are one of their manifestations (Behcet's syndrome), smokers, and individuals who could not continue the study due to personal or social reasons, were excluded [13].

### Developing the pharmaceutical product

10-mg cinnamaldehyde mucoadhesive tablets were developed with different proportions of adhesive polymers (such as Carbopol HPMC K4M) alone and mixed. The pharmaceutical drug and polymer were mixed. After complete mixing, the magnesium stearates were added, and the tablets were made using direct compression. Evaluation of changes in weight, hardness, erosion, tensile strength, and value determination were measured by spectrophotometry. The number of active compounds in the tablets after 0.5, 1, 1.5, 2, 3, and 4 h was determined by ultraviolet spectrophotometry in at least three samples.

The mucoadhesive strength of the evaluated formulations was determined using a sodium alginate gel; to do so, a spool and a string attached to the tablet were used. After the tablet touched the gel surface, the amount of force needed to separate the tablet from the gel surface was measured by pouring water into the bottom of the string container. After calculating the weight force applied to detach the tablet, the weight was multiplied by the gravity acceleration. Based on the tablet surface area, the adhesion force per area unit was determined in N/m^2^.

The present study is a continuation of an animal study conducted at Mazandaran University of Medical Sciences [24], in which the safety and efficacy of cinnamaldehyde were examined on Wistar rats. In the present study, cinnamaldehyde 98% was purchased from Merck–Germany. Based on the quality approvals as well as the approval of the ethics committee of Mazandaran university of medical sciences, the authors examined cinnamaldehyde patches on humans in the present study.

It should be noted that each patient was assured about the safety of the cinnamaldehyde patches before they were enrolled in the present study and was informed about the previous study that was performed on rats [24]. After the completion of the clinical trial, none of the patients showed any particular side effects or allergies to the cinnamaldehyde patches.

### Study protocol

The examiner and the patients were blinded. All patients in the two groups were blinded and asked to refer to the clinic within the first 24 h after the appearance of the aphthous lesions. This period was considered as the baseline (day zero). In the first group, patients were taught how to use three mucoadhesive patches and were asked to use them daily in the morning, afternoon, and night. Each individual was asked to avoid eating and drinking for 30 min after using the patches. In the control group, the same was done with a placebo. Patients were clinically examined on days 0, 3, 5, and 7 to evaluate the pain intensity and healing of the ulcers. The examiner was blinded and was unaware of the medication each patient was receiving. Metal calibers were used to determine the diameter of the ulcers and the surrounding inflammatory zone (by millimeters) [13]. Patients were also asked to select the pain intensity according to the Visual Analogue Scale (VAS) criteria. This scale consists of a 10-cm line in which zero indicates no pain and ten indicates the maximum pain. The patient marked a point representing their pain on this scale and used the numeral scale (e.g., from one to ten) to estimate the pain intensity. Patients were asked to report the VAS 3 times a day after each meal. Usually, pain stimulation in the oral cavity reaches its maximum after mechanical and chemical stimulation. Eating and chewing stimulate the oral mucosa by mechanical stimuli caused by the presence of food particles as well as chemical stimuli derived from the presence of acids, spices, and salt in the food. Therefore, the measurement of pain during meals can accurately reflect the overall pain of the oral mucosa. Patients with a pain score of 1 and ulcer size of less than 1 mm were considered to be healed [25].

### Data analysis

Data analysis was performed using the SPSS-20 statistical software; notable that the analyzer was blinded. Variables were described by percentage, mean and standard deviation. Fisher's exact test was used to compare the gender variable between the two groups. The mean age was compared between the two groups using an independent sample t-test, and quantitative variables of normal distribution were evaluated using the Shapiro Wilk test. The Friedman test was used to examine the time intervals in the two groups, and the Mann–Whitney test was used to compare the two groups at different measurement times. The Generalized Estimation Equation (GEE) test was used to compare the time trend of changes in the inflammatory zone diameter and pain intensity between the two groups.

## Results

**Fig. 1:**
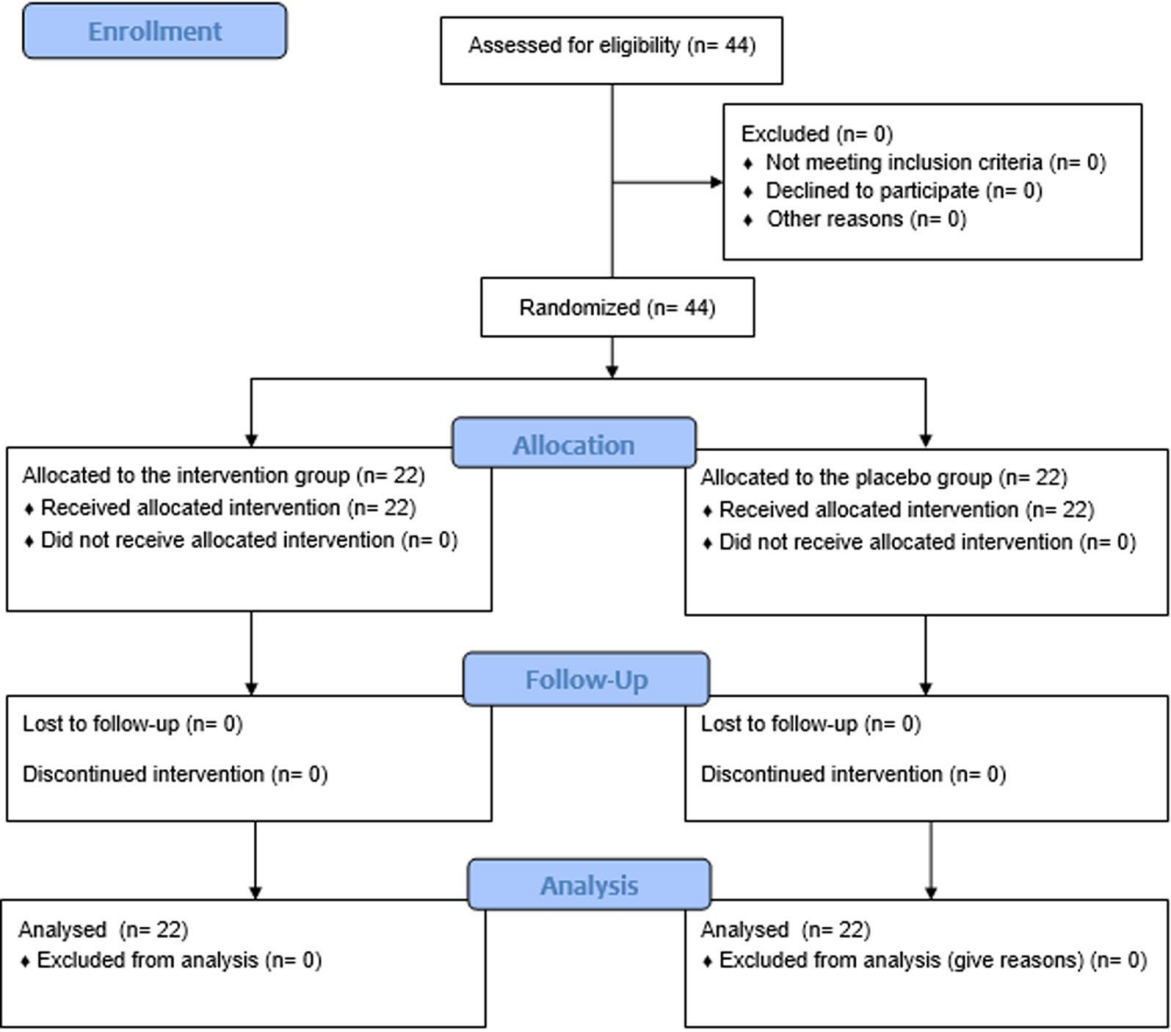
Diagram of patient enrollment and study process

This double-blind, randomized clinical trial was conducted on forty-four patients randomly divided into the intervention (Cinnamaldehyde) and control (placebo) groups. Each group consisted of 22 patients. No side effects were observed in any group during the intervention. (Fig. 1).

Gender distribution in the two groups did not show a statistically significant difference (*P* = 0.698). Also, the mean age in the two groups was not statistically significant (Table 1).

**Table 1:** Mean ± SD of age and gender in the two study groups

Variable		Cinnamaldehyde Group [22]	Placebo Group [22]	*P*-Value
Gender	`Male	3(%13.6)	5(%22.7)	0.698
	Female	19(%86.4)	17(%77.3)	
Age (years)	Mean ± SD.`	30.59 ± 6.08	30.41 ± 5.41	0.918

The period of the intervention was seven days. The diameter of the aphthous ulcer (by millimeters) was evaluated during days 0, 3, 5, and 7. On day 0, the diameter of the inflammatory zone was not statistically significant in the two groups. But on the third, fifth, and seventh days of the study, the diameter of the lesion showed a statistically significant difference between the two groups (*P* < 0.001). The decreasing trend of the erythematous zone diameter in the cinnamaldehyde group was higher than in the placebo group. The trend of intergroup and intragroup changes in inflammatory zone diameter overtime was also statistically significant (*P* < 0.001).

The pain intensity after meals in each group showed a statistically significant decrease from day 0 to day 7. In intergroup comparison, changes in pain intensity from the first day (*P* = 0.024) to the seventh day (*P* < 0.001) of the study were statistically significant. Generally, the trend of pain intensity changes over time between the two groups was statistically significant (*P* = 0.001) (Table 2).

**Table 2:** Mean ± SD of ulcer size and pain intensity during the study days in the two groups

Variable	Days	Group		*P*-value	*P*-value
		Cinnamaldehyde	Placebo		
Ulcer size (millimeters)	0	4.4 ± 1.5	4.5 ± 1.47	0.666	< 0.001*
	3	2.27 ± 1.35	4.8 ± 1.33	< 0.001 *	
	5	0.59 ± 0.95	2.81 ± 1.65	< 0.001 *	
	7	0.04 ± 0.21	1.5 ± 1.26	< 0.001 *	
	*P*-value	< 0.001 *	< 0.001 *		
Pain intensity (after meals)	0	6.77 ± 2.18	8.0 ± 1.34	0.07	< 0.001*
	1	5.5 ± 2.62	7.17 ± 1.35	0.024 *	
	2	3.79 ± 2/32	7.02 ± 1.71	< 0.001 *	
	3	2.08 ± 1.86	6.1 ± 1.8	< 0.001 *	
	4	0.98 ± 1.28	4.98 ± 2.18	< 0.001 *	
	5	0.32 ± 0.74	3.97 ± 2	< 0.001 *	
	6	0.0 ± 0.0	2.43 ± 1.72	< 0.001 *	
	7	0.0 ± 0.0	1.21 ± 1.28	< 0.001 *	
	*P*-value	< 0.001 *	< 0.001 *		

In the cinnamaldehyde group, the mean pain intensity (after meals) on the fourth day was 0.98 ± 1.28, and on the seventh day, the mean pain intensity decreased to zero. In contrast, despite a significant decrease in pain intensity in the placebo group, the mean pain intensity was reported as 1/21 ± 1/28 until the seventh day of the study.

## Discussion

The present study investigated the effect of Cinnamaldehyde mucoadhesive patches on recurrent minor aphthous stomatitis. According to the results, the size of the aphthous ulcers in the cinnamaldehyde group was significantly reduced compared to the control group. According to the mentioned criteria, the aphthous lesions in the intervention group were considered improved on the fifth day. The mean diameter of the inflammatory zone of the placebo group was more than 1 mm on the seventh day, and no complete improvement was observed in the aphthous lesions of this group. The pain intensity was significantly different between the two groups during the study days except for baseline. The decreasing pain trend was higher in the cinnamaldehyde group than in the placebo group. The mean pain intensity in the intervention group was less than one on the fourth day. Painless conditions were reported in the intervention group on the sixth and seventh days of the study. While in the placebo group, even on the seventh day of the study, the mean pain intensity was higher than 1.

Recurrent aphthous stomatitis is a common disorder of the oral cavity diagnosed by pain and inflammation of the oral mucosa. Due to the unknown etiology of the lesion, a definitive treatment was not obtained for RAS due to its nonspecific treatment. The most important therapeutic goals are to alleviate pain and disability and reduce inflammatory responses and recurrences of the lesion [10]. The anti-inflammatory, antioxidant and antibacterial effects of Cinnamaldehyde include decreased production of prostaglandin E2 and inhibition of cyclooxygenase, two expressions (COX-2). As a result, a significant reduction in interleukin-1-beta increased glutathione peroxidase enzyme activity, and bactericidal properties against gram-positive and gram-negative bacteria are among the results of previous studies [24].

Muhammad et al*.* reported that Cinnamaldehyde significantly reduces the production and secretion of interleukin 8. Also, it prevents the activation of NF-KB [26]. Molania et al. studied the effects of Cinnamaldehyde on mucositis and salivary antioxidant capacity in gamma-irradiated rats. The mean mucositis incidence in irradiated rats in the cinnamaldehyde group was lower than in the control group (normal saline). They attributed the cause of this result to the anti-inflammatory, antibacterial and antioxidant effects of Cinnamaldehyde [24].

In a study by Molania et al*.,* the anti-inflammatory effects of Cinnamaldehyde mouthwash in patients with gingivitis showed no statistical significance compared to chlorhexidine. They suggested that Cinnamaldehyde can be considered as a herbal mouthwash for improving the gingival condition without the side effects of chlorhexidine [27]. Similar clinical trials conducted on the same topic in human patients suggest that oral mucosa adhering agents can be considered as the first-line treatment for minor aphthous ulcers. Non-pharmaceutical products are reliable sources for the appearance of new therapeutic approaches due to their fewer side effects and higher patient acceptance and desire. Applying a coating on aphthous ulcers considerably reduces the patient’s pain, improves the healing, and eliminates the need for patients to use antibiotics or any other form of the drug [28].

Yao et al. Conducted a study on the effect of Cinnamaldehyde on stress in rats. They stated that cinnamaldehyde extract has antidepressant effects on stress conditions in middle-aged rats by reducing the amount and activity of cyclooxygenase, two proteins [15]. According to the former relevant studies, it seems that a significant reduction in the size of the aphthous lesion and the severity of pain in patients of the cinnamaldehyde group compared to the placebo group is related to the anti-inflammatory and analgesic or antioxidant properties of Cinnamaldehyde in the present study. According to the results of the present study, mucoadhesive patches showed ease in use and also short-term pain relief in patients with RAS, which attracted more patient satisfaction and cooperation and resulted in better therapeutic effects.

## Conclusion

Cinnamaldehyde mucoadhesive patches effectively reduced and improved aphthous lesions and pain intensity in patients and can be considered as a treatment for RAS.

### Strength and limitations

We believe that this was the first study to examine the impact of Cinnamaldehyde mucoadhesive patches on aphthous ulcers. Therefore, the lack of similar studies due to the novelty of the subject is one of the main limitations of the present study. It is recommended to conduct more studies with a larger sample size to achieve more reliable results. Also, the lack of supervision and direct access to patients during the seven days of the stud can be listed as another limitation of the present study.

## Data Availability

The datasets used and/or analyzed during the current study are available from the corresponding author on reasonable request.

## Abbreviations

RAS: Recurrent aphthous stomatitis
ROS: Reactive oxygen species
VAS: Visual analogue scale
